# Learning in a crisis moment: a randomized controlled trial in emergency bystander intervention

**DOI:** 10.1186/s40359-023-01146-x

**Published:** 2023-07-21

**Authors:** Caitlin McGaffick, Noor Gulrajani, Nan Kong, Nicole Adams

**Affiliations:** 1 School of Nursing, Purdue University, West Lafayette, USA; 2 Weldon School of Biomedical Engineering, Purdue University, West Lafayette, USA; 3 Johnson Hall of Nursing, 502 N University St, West Lafayette, IN 47907, 765-494-4025 USA; 4 Johnson Hall of Nursing, 502 N University St, West Lafayette, IN 47907, 260-449-0316 USA

**Keywords:** Working memory, Instantaneous learning, Bystander training, Emergency response, Crisis communication, Community health, Acute stress

## Abstract

**Background:**

Opioid overdose is the leading cause of injury-related death in the United States. Individuals who overdose outside of clinical settings have more positive clinical outcomes when they receive naloxone, an opioid antagonist, from a bystander as an early intervention before emergency personnel arrive. However, there is a gap in knowledge about successful instantaneous learning and intervention in a real-life stressful environment. The objective of this study is to explore the efficacy of different instructional delivery methods for bystanders in a stressful environment. We aim to evaluate which methods are most effective for instantaneous learning, successful intervention, and improved clinical outcomes.

**Methods:**

To explore instantaneous learning in a stressful environment, we conducted a quantitative randomized controlled trial to measure how accurately individuals responded to memory-based survey questions guided by different instructional methods. Students from a large university in the Midwest (*n* = 157) were recruited in a public space on campus and accessed the six-question survey on their mobile devices. The intervention group competed the survey immediately while the research team created a distracting environment. The control group was asked to complete the survey later in a quiet environment.

**Results:**

The intervention group correctly answered 0.72 questions fewer than the control group (*p* = .000, CI [0.337, 1.103]). Questions Q1 and Q5 contained direct instructions with a verbal component and showed the greatest accuracy with over 90% correct for both stressful and controlled environments.

**Conclusions:**

The variability in the responses suggests that there are environmental factors as well as instructional design features which influence instantaneous learning. The findings of this study begin to address the gap in knowledge about the effects of stress on instantaneous learning and the most effective types of instruction for untrained bystanders in emergency situations.

## Background

Drug overdose is the leading cause of injury-related death in the United States, with 70% of those deaths attributed to opioids [1]. In the event of an overdose occurring outside of a medical setting, bystanders have an important role in contacting emergency medical services and can provide basic care to the victim before help arrives. Naloxone, an opioid antagonist, is the reversal agent that should be administered as quickly as possible to a victim suspected of an opioid overdose. Patients who receive naloxone from a bystander as an early intervention to opioid overdose have more positive clinical outcomes [2]. Naloxone will not harm a victim who does not have opioids in their system [3].

Doe-Simkins et al. [4] displayed the successful reversal of opioid overdose through the administration of naloxone by trained bystanders in a controlled environment. However, there is little research about how a stressful environment impacts the ability of bystanders to accurately follow naloxone administration instructions. In this study, we explore the effects of a stressful environment, when compared to a controlled environment, on the ability of bystanders to accurately answer memory-based survey questions. The objective of this study is to explore the efficacy of different instructional delivery methods for bystanders in a stressful environment compared to a controlled environment. We aim to gain insight about the type of instructions that will be most effective for a medically naïve bystander to successfully administer naloxone to an opioid overdose victim. Our findings are intended to contribute to knowledge and guide the development of a medical interventions for untrained bystanders.

### Bystander intervention and working memory

Bystanders who voluntarily intervene in a potentially life-threatening event such as a heart attack, drug overdose, or allergic reaction have a substantial opportunity to improve the survival rates of victims with their early assistance [5]. Unfortunately, emergency medical services (EMS) providers reported bystander assistance in only 11% of medical emergencies [6].

Various situational and personal factors determine whether a bystander will take action in an emergency [6]. Kagan [7] showed that bystanders are more ready and willing to act if they are familiar with the emergency situation through special training, personal experiences, or general knowledge. To provide optimal support and ensure the efficacy of bystanders that do take action, it is important to determine how the stressful environment of an emergency situation may play a role in a bystander’s active response to instructions they are given when attempting to assist a victim before professional help arrives.

Unexpected medical dangers, time pressures, and social threats associated with bystander intervention are likely to create a stressful situation for bystanders. Stress initiates a series of physiologic reactions in the body which are manifested as the “fight or flight” response [8]. This sympathetic state increases cortisol and dopamine levels, which alter the way that the prefrontal cortex responds to reinforcement learning [9]. However, whether these changes result in positive or negative outcomes is highly individualized. Using a probabilistic learning task associated with positive or negative feedback, Lighthall [9] found that learning is improved by feedback that predicts a positive outcome. In contrast, de Berker et al. [10] used instrumental learning, or operant conditioning, to conclude that stress may impair the ability to actively respond to reinforcement. Due to individualization, learning by reinforcement is unlikely to be the most effective technique for delivering instructions to bystanders. Little is known about effective strategies for instantaneous learning among the general public.

Working memory may play a role the ability to follow instructions. To increase the probability of properly following instructions, Dunham et al. [11] recommended that learners are (1) provided with instructions in the order they must be completed, (2) teaching back the instructions, and (3) allowing the learner to control the rate at which the instructions are presented. These recommendations address the complications of limited working memory and may improve adherence to instruction [11]. In a medical emergency, these steps may be less individualized and more beneficial than reinforcement methods for promoting short-term learning and facilitating a favorable bystander response to instructions.

The Model of Working Memory [12] holds that the phonological loop and the visuospatial sketchpad are two separate components of working memory that interact with the central executive in order to learn instantaneously. It is also known that reading and visualizing require the same neural pathways, so auditory instructions are more accurately visualized and actively followed than written instructions [13]. This also means that if the information and task draw on the same neural pathway of working memory, then performance is worse [14]. Therefore, we know that working memory is better when different types of information are presented simultaneously. These fundamental concepts assisted in our methodology, specifically in the types of questions we developed with varying degrees of audio and visual components [11]. However, little is known about how distraction and stress affect working memory and learning of a bystander in an emergency situation. This knowledge is essential for the creation of effective instructions provided to bystanders during medical emergencies.

### AED

Our interest is in bystander administration of Narcan, which has not been well studied. Most of the existing research on bystander emergency response is related to automated external defibrillators (AEDs). An AED is the leading emergency technology used for cardiac arrest. Bystander training in cardiopulmonary resuscitation (CPR) and AEDs is common and typically performed on manikins in controlled environments. The fundamental components of bystander training identified in AED research were used to inform the methodology of our study.

The majority of untrained laypeople are not able to effectively operate an AED, despite its intention to be usable by all people in emergencies [15]. In a simulation study, Andre et al. [16] found that the AED user interface made a significant impact on the ability of lay bystanders to accurately respond to instructions. The most effective AED design included specific voice prompts associated with intuitive visual diagrams [16]. Deficits in bystander performance were attributed to AEDs which (a) must be manually turned on, (b) provided minimal or implicit instructions, (c) involved components that may become loose or detached, (d) did not provide an image for pad placement, or (e) failed to explicitly guide the user through the appropriate steps [16]. The given deficits relate directly to how effectively the AED design makes use of the bystander’s working memory.

Likewise, Biancardi et al. [17] found that direct phrases in verbal telephone instructions were most effective for directing lay bystanders to perform CPR and use an AED. Direct phrases leave little room for personal interpretation, unlike implicit phrases associated with the subject’s willingness to act. Rudland et al. [8] concluded that verbal instructions supported the bystander in tuning out personal interpretations and environmental stressors.

When providing instructions, it is important to avoid cognitive overload for the bystander. Even if direct verbal instruction is used, the prompt may not be effective if it is accompanied by complex videos or diagrams. Ettl et al. [18] found that audio-video instructions did not significantly improve the ability of laypeople to follow AED instructions due to cognitive overload, although participants reported feeling more support with an additional video. The visual aspect of learning must not be dismissed, as Yang [19] found that written or visual instructions were more beneficial in producing an accurate response from the working memory when compared to verbal instructions. There must be a balance of verbal and visual instructions for optimal bystander intervention. Accordingly, a bystander of a medical emergency should be guided by direct, orderly instructions with an intuitive visual component to elicit the best possible outcome for the victim. The insight gained from studying AED instructions are undoubtedly beneficial; however, there remains a gap in knowledge about instantaneous learning in real-life stressful environments as compared to controlled environments, which may have an impact on how instructions are presented to bystanders of opioid overdose.

## Methods

### Study design

This study was a randomized controlled trial using a single survey to deliver instructions and collect response data. All data was collected via electronic survey without any identifiers. Although researchers were not blinded as they created the intervention environment, they were also not aware of the responses of the participants as they completed the surveys.

### Participants

The Purdue University Human Research Protection Program/Institutional Review Board (IRB) granted the present study an exemption for face-to-face human subject research on March 3, 2021. In compliance with COVID-19 protocols, participants were recruited on Krach Lawn, a popular outdoor area at a large university in the Midwest. Social distancing was maintained, and masks were worn by the research team during active participation.

Participants were limited to currently enrolled students of any level. For this study we used college students as a proxy for untrained bystanders, and the survey we created was new information to all of them. All participants who approached the table with interest in the study were provided with an information sheet, which contained the information usually presented in a consent form. Signed consent was not obtained as it would have been the only identifiable information collected from participants. Implied informed consent was provided through completion of the survey and was obtained from all participants and/or their legal guardians. Enticement such as candy, music, and colorful posters were used to attract participants. Non-personally identifiable information was collected at the end of the survey, including gender, race/ethnicity, age, year, and major.

### Interventions

Distractions were set up to create a stressful environment while participants in the intervention group were taking the survey. These distractions, which were both COVID-19 protocol adherent and innocuous, included street traffic, popping balloons, loud music, students playing games in the lawn, phone alarm interruptions, and absurd lightsaber battles (Star Wars style). The distractions were designed to create a disturbing, irritating environment that simulated stress in a real-world scenario.

### Sample size

Preliminary tests were performed to find approximate values for power analysis. Eight undergraduate classmates were asked to test the survey: three of which were in a somewhat stressful environment and five of which were in a relaxed, quiet environment. From the results of this preliminary test, two hypotheses were generated: the null hypothesis, which stated that on average there is no difference in the number of questions answered correctly between the control group and intervention group, and the alternative hypothesis, which stated that on average, the intervention group would answer one fewer question correctly. The alternative hypothesis was developed from the preliminary testing, which showed that those who took the survey in a stressful environment answered 3.67 questions correctly, whereas those who took the survey in a calm environment answered 4.40 questions correctly. The effect size of 0.73 was rounded up to 1 whole question for the alternative hypothesis.

A priori power analysis was performed to estimate the sample size [20], based on our preliminary data. With an alpha of 0.05 and power of 0.80, the estimated sample size needed with an effect size of 0.73 is 60 participants. Because our effect size is relatively large according to Cohen’s conventions [21], our projected sample size was increased to 150 total participants, approximately 75 in each group.

After three days of outdoor data collection, a sufficient sample size for the intervention group (*n* = 78) was attained. However, participant adherence in the control group was lower than expected (*n* = 34), despite handing out more surveys. To increase the sample size for the control group, the survey and the information sheet were sent to groups of students via GroupMe messaging. Four different group chats containing 335 people total were involved. All group chats consisted of a variety of students, across years and majors of undergraduate degrees. After participants from GroupMe recruitment responded, the total control group sample size was sufficient (*n* = 79). All methods were carried out in accordance with relevant guidelines and regulations.

### Randomization

Each participant was provided a piece of paper with both a QR code and a web address link to the survey. By scanning the QR code or entering the link into an internet browser, participants could access the survey on their own devices and adhere to COVID-19 safety protocols. The intervention and control groups both completed the same questions in the same order, but they were recorded in separate surveys to allow for comparison.

Intervention and control QR codes and instructions were mixed and handed out in the order in which they were stacked, creating randomization. The intervention group was instructed to scan the QR code and begin taking the survey immediately. The control group was instructed to take the survey in the next 24 h at their leisure in a quiet environment. Anticipating drop out among the control group (forgetting to complete it once at home), more control group instructions were distributed (128 control, 78 intervention).

### Survey design

**Fig. 1:**
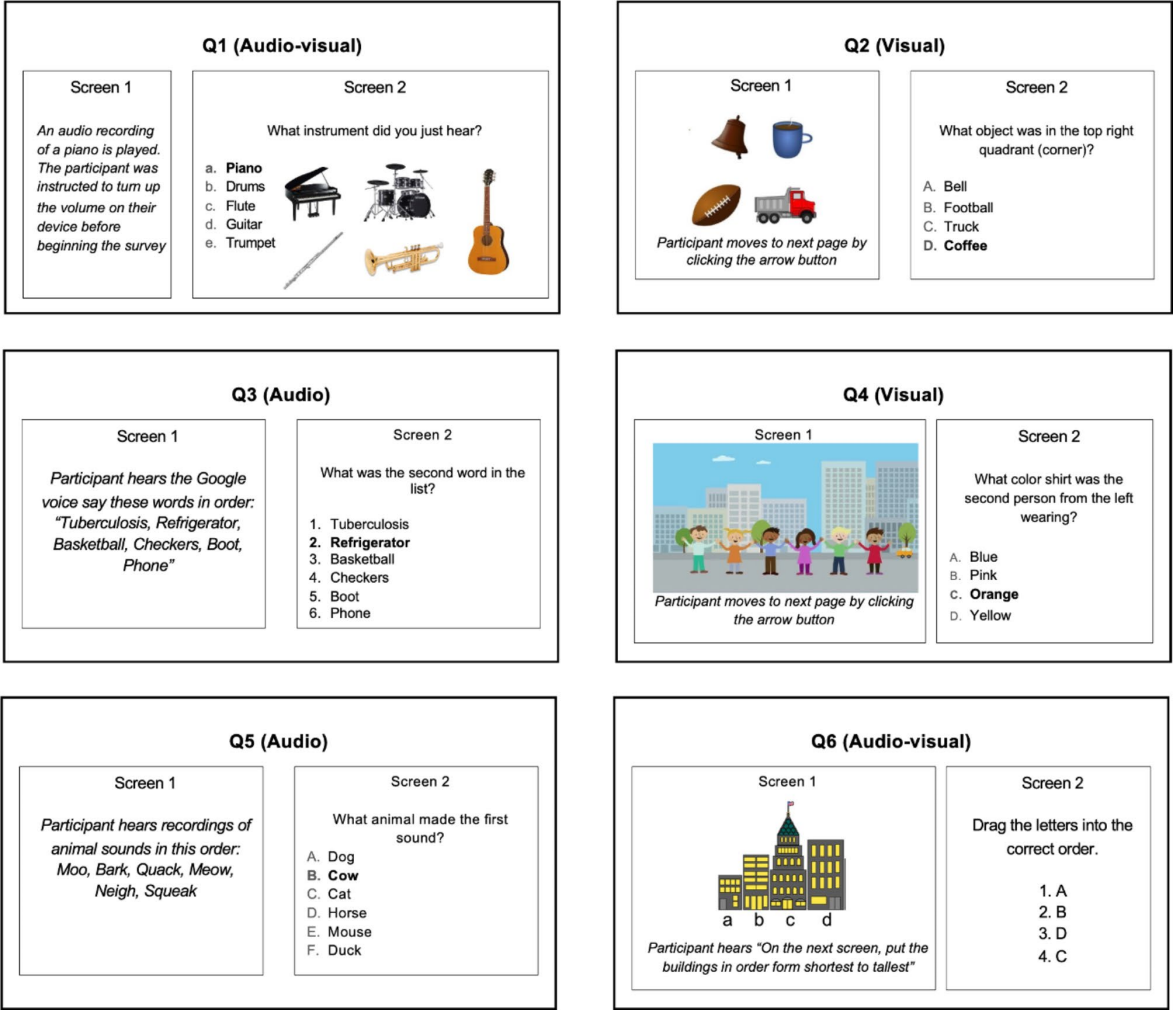
Survey Questions

Six memory questions with different combinations of audio and visual components were integrated into a Qualtrics online survey to test the ability of the working memory to respond accurately in a stressful versus controlled environment. All participants received the same questions in the same order. The survey questions are available in Fig. 1.

Two questions tested the efficiency of audio-only instruction (Q3 and Q5) by providing an audio sample of random words or sounds with no context. The next screen targeted the working memory with a question about the audio content where the participant selected their answer from a list of multiple-choice options. Two questions tested the efficacy of visual-only instruction (Q2 and Q4) by providing a picture on the first screen with no additional instruction and a memory question with a list of multiple-choice options on the next screen. Participants selected an arrow button to move to the second screen, and they were not able to return to the previous screen at any time. Two questions tested a blend of audio and visual components (Q1 and Q6). One question verbally asked the participants to place the pictured four buildings in order from shortest to tallest (Q6), and the other asked participants to identify the picture of the instrument that they heard in the audio clip (Q1).

### Statistical analysis

Data was extracted from Qualtrics into Excel files where it was cleaned and prepared by checking for missing inputs and transforming variables. The primary outcome measure was accuracy of answering the survey questions.

Data analysis was conducted using SPSS version 26. Descriptive statistics included frequencies, crosstabs, and chi-square test for both demographics and response accuracy. Independent sample t-tests were conducted to analyze the difference in overall response accuracy between groups. A summary of this study was presented as an interdisciplinary project in the 2021 Purdue University Undergraduate Research Conference and is available to the public on YouTube [22]. This study was not preregistered.

## Results

The sample demographics compared to the University student population are presented in Table 1. The overall sample was representative of the student population at large for age, major, and gender (*p* > .05), but not for year and race. The demographics of the control and intervention groups were significantly different for gender and race (Table 2) with more Caucasians and females in the control group. Engineering was the most prominent major represented, which was similar between the two groups.

**Table 1:** Study participants compared to the University population (χ²)

		Sample (*n* = 157)				University Population				Pearson χ^2^ value	*p-*value (Pearson χ^2^*)*
		Frequency		Percentage		Frequency			Percentage		
Age										3.965	0.265
	**18–19**	87	55.8%			14,031		42.7%			
	**20–21**	53	34.0%			13,703		41.7%			
	**22–24**	14	9.0%			4481		13.6%			
	**25–29**	2	1.3%			632		1.9%			
	**Total**	156	100%			32,847		100%			
**Major**										**4.695**	**0.860**
	**Engineering**	61			38.9%	13,761	30.9%				
	**Science**	18			11.5%	6422	14.4%				
	**Health & Human Sciences**	16			10.2%	5504	12.4%				
	**Polytechnic**	16			10.2%	5045	11.3%				
	**Liberal Arts**	14			8.9%	3211	7.2%				
	**Management**	12			7.5%	3211	7.2%				
	**Pharmacy**	8			5.1%	1376	3.1%				
	**Agriculture**	5			3.2%	3211	7.2%				
	**Exploratory**	5			3.2%	1376	3.1%				
	**Education**	2			1.3%	1376	3.1%				
	**Total**	157			100%	44,493	100%				
**Year**										**20.454**	**< 0.001**
	**Freshman**	63	41.4%			7785	22.4%				
	**Sophomore**	51	33.6%			7558	21.8%				
	**Junior**	18	11.8%			7902	22.8%				
	**Senior**	20	13.2%			11,491	33.1%				
	**Total**	152	100%			34,736	100%				
**Gender**										**0.504**	**0.478**
	**Male**	81	52.3%			26,057	56.8%				
	**Female**	74	47.7%			19,812	43.2%				
	**Total**	155	100%			45,869	100%				
**Race**										**41.466**	**< 0.001**
	**Caucasian**	97	63.0%			35,693	78.1%				
	**Asian**	35	22.7%			4358	9.5%				
	**Hispanic or Latino**	9	5.8%			2660	5.8%				
	**Multiracial**	8	5.2%			1689	3.7%				
	**African American/Black**	4	2.6%			1284	2.8%				
	**Native Hawaiian/ Pacific Islander**	1	0.6%			30	0.1%				
	**Total**	154	100%			45,714	100%				

**Table 2:** Comparing demographics of the control and intervention groups (χ²)

		Crosstabs		Pearson χ^2^ value	*p-*value (Pearson χ^2^*)*
		**Intervention** (*n* = 78)	**Control** (*n* = 79)		
Age				**5.874**	**0.555**
	**18**	12	12		
	**19**	31	32		
	**20**	15	21		
	**21**	12	5		
	**22**	5	5		
	**23**	1	1		
	**24**	1	1		
	**25**	0	2		
Gender				**16.845**	**0.000**
	**Male**	53	28		
	**Female**	24	50		
	**Other**	1	1		
Major				**12.252**	**0.199**
	**Agriculture**	3	2		
	**Education**	1	1		
	**Engineering**	29	32		
	**Exploratory**	2	3		
	**Health & Human Sciences**	6	10		
	**Liberal Arts**	4	10		
	**Management**	9	3		
	**Pharmacy**	2	6		
	**Polytechnic**	11	5		
	**Science**	11	7		
Year				**5.620**	**0.467**
	**Freshman**	32	31		
	**Sophomore**	21	30		
	**Junior**	11	7		
	**Senior**	12	8		
	**5th Year Senior**	0	1		
	**Graduate Student**	2	1		
	**Other**	0	1		
Race				**18.863**	**0.004**
	**African American/ Black**	4	0		
	**Asian**	22	13		
	**Caucasian**	36	61		
	**Hispanic/Latino**	7	2		
	**Multiracial**	6	2		
	**Native Hawaiian/ Pacific Islander**	1	0		
	**Other**	2	1		

The number of survey questions answered correctly by the control group ranged from 2 to 6, with a mean of 4.85 (*SD* = 1.10). The accuracy in the intervention group ranged from 0 to 6 questions correct, with a mean of 4.13 (*SD* = 1.32). There is a mean difference of 0.72 between the two groups, which is statistically significant (*p* = .000, CI [0.337, 1.103]). The variability in the responses suggests that there are environmental factors as well as instructional design features which influence instantaneous learning.

The comparison between groups of the total number of questions answered correctly is shown in Table 3. The number of control group participants who accurately answered all six questions (*n* = 26) is double the number of intervention group participants with the same accuracy (*n* = 13).

**Table 3:** Frequency of accurate responses by group

	Number Correct	Frequency	Percentage
**Control** (*n* = 79)	5	28	35.4%
	6	26	32.9%
	4	15	19.0%
	3	7	8.9%
	2	3	3.8%
	Total	79	100%
**Intervention** (*n* = 78)	4	26	33.3%
	5	17	21.8%
	3	15	19.2%
	6	13	16.7%
	2	5	6.4%
	0	2	2.6%
	Total	78	100%

The accuracy of responses by question in each group is shown in Table 4. Questions 1 and 5 were consistently answered correctly by both groups, with the control group being slightly more accurate but without statistical significance (*p* = .142 for Q1 and *p* = .113 for Q5). Question 3 was answered far less accurately by both groups, with the control group remaining slightly more accurate but the difference was not statistically significant (*p* = .226). The control group was significantly more accurate in answering Questions 2 and 6 than the intervention group (*p* < .05). The intervention group was slightly more accurate in answering question 4 than the control group, but the difference was not statistically significant (*p* = .947).

**Table 4:** Frequency of correct responses by question

	Intervention (*n* = 78)		Control (*n* = 79)		*P* value (Pearson χ^2^)	Pearson χ^2^ value
	Freq. Correct	% Correct	Freq. Correct	% Correct		
**Question 1** *Audio-visual*	72	93.5%	76	97.4%	0.142	2.162
**Question 2** *Visual*	40	54.4%	60	76.9%	0.001	10.327
**Question 3** *Audio*	44	58.4%	52	66.6%	0.226	1.464
**Question 4** *Visual*	46	62.3%	47	60.3%	0.947	0.004
**Question 5** *Audio*	70	92.2%	76	97.4%	0.113	2.513
**Question 6** *Audio-visual*	51	66.2%	71	91.0%	0.000	14.750

## Discussion

To optimize the ability of a bystander to accurately follow instructions in a stressful environment, the mode of instruction must be tailored to the working memory function under stress. Our results show that an audio component is most effective when it is phrased directly and/or appears in conjunction with visual instruction. Questions Q1 and Q5 displayed the most accurate responses for both intervention and control groups, with 97.4% accuracy for both questions in the control group and the 93.5% and 92.9% accuracy on Q1 and Q5 respectively in the intervention group (Table 4). Q1 included direct instructions with an audio component and straightforward visual clip art, and Q5 audio-only. This suggests that instructions modeled in a style similar to Q1 and Q5 can negate the impact of stress and distraction on the ability to learn a new task.

There must be a balance of audio and visual output due to the possibility of cognitive overload [18] which may explain the results for question Q6. This question required the participant to associate a certain building height with a letter (a, b, c, d), and then to put the letters in order of shortest to tallest building on the next page. It was the only survey question that required objects to be placed in a specific order. The instructions on the first screen were provided verbally but were not written out visually. The control group displayed 91.0% accuracy for question six while the intervention group displayed only 66.2% accuracy (*p* < .05), indicating that a stressful environment has a significant impact on instantaneous learning for this instructional method. The results for Q6 provide an example of instructions that are too complex for bystanders in a stressful environment to utilize effectively. Instructions of this nature may lead to cognitive overload and prevent a bystander from successful intervention. Although Q1 and Q6 were both audio-visual questions, they differed significantly in difficulty. Q1 provided the proper balance of direct audio-visual instructions, while Q6 provided complex information that led to cognitive overload when performing the task in a stressful environment. In future application with bystanders, we suggest audio-visual instructions be structured more directly as they were in Q1, with fewer simultaneous tasks for the user to learn. Direct instructions given one at a time may reduce cognitive overload and therefore may be more likely to be followed accurately by a bystander under acute stress.

The control group did significantly better in accuracy on the visual-only Q2 than the intervention group, with 76.9% accuracy in the control group and 54.4% accuracy in the intervention group (*p* < .05). This suggests that stress and distraction were a significant factor in the ability to recall the images. However, there is no statistically significant difference between the groups on the other visual-only question Q4. Both groups answered Q4 with very low accuracy: 60.3% accuracy in the control group and 62.3% accuracy in the intervention group. We postulate that this low accuracy may be due to cognitive overload created by the image in Q4 as it was far more complex in detail than the image in Q2, resulting in participants not focusing on the detail they needed to recall regardless of environment (Fig. 1).

In a similar context, the audio-only question Q3 was overall lower in accuracy than audio-only Q5. Results for Q3 showed 66.6% accuracy in the control group and 58.4% accuracy in the intervention group, compared to 97.4% accuracy in the control group and 92.2% accuracy in the intervention group for Q5. The instructions in Q3 asked the participant to identify the second word that they heard from a list of random words, while Q5 asked the participant to identify the first sound that they heard from a series of animal noises. Because the content of both questions would be considered generally familiar and simple, we do not suspect the specifics of the words or sounds to be at play in the accuracy discrepancy between questions. Rather, we postulate that the primacy effect [23] may be at play, further emphasizing the efficacy of direct instructions that provide the most important information first.

Our study finds that a stressful environment negatively impacts the ability of the working memory to respond accurately to instructions. While this was expected, the variety of instruction methods used in our study implies that certain types of instructions provided to bystanders are more likely to improve outcomes for opioid overdose victims. Administrating naloxone to victims is likely to be impacted by the inevitably stressful environment of a medical emergency, so the instructions for bystanders must be effective. Only half the number of participants in the intervention group followed instructions with 100% accuracy when compared to the control group. By extension, if only half of the bystanders who receive instructions are able to successfully intervene, then the instructions are not effective enough to maximize clinical outcomes of overdose victims. Therefore, we recommend that instructions presented to bystanders are direct, containing a balance of audio and visual components to avoid cognitive overload in stressful environments. Important information should be clearly stated with one task to perform at a time.

### Limitations

Our study does have several significant limitations related to sample. In addition to a small sample size, there were statistically significant differences between the demographics of our sample and the University’s general student population in respect to year and race (Table 1). Individuals who were freshmen were overrepresented in the present study when compared to the University demographics. This is likely due to the recruitment location being in close proximity to underclassman-dominant housing and dining on campus. Additionally, the Caucasian race was overrepresented, and the Asian race was underrepresented in the present study when compared to the University demographics. Gender and race differed significantly between the intervention group and control group, as the control group was comprised of more Caucasians, and the intervention group was predominantly comprised of males. The variability in demographics may be due to differences in confidence and cultural norms in approaching a public table. Furthermore, we were unable to completely compare gender and year because data for these demographics for students who identify as “third gender/non-binary” for gender, “other” for race, and “5th year senior” or “other” for year, were not available at the University level. The University data for year was classified by number of credit hours; however, students often state their year in their program rather than by credit hours. This leaves the possibility of a biased sample through size and composition, and further studies are indicated to replicate findings.

## Conclusions

We believe that our simulation of a stressful environment for the intervention group was effective for showing a difference in instantaneous learning depending on environment. There is a substantial body of knowledge related to teaching and learning methods, although there is lacking knowledge on evaluating learning in a high stress environment and then applying the learning immediately to a task. Once learning modules are developed to train bystanders how to intervene in an emergency situation and tested for usability in controlled environments, they could then be tested in stressful simulations using the methods described here. Consistent with educational psychology, instructions given one at a time with a balanced combination of visual and audio cues resulted in highest accuracy responses.

This study contributes both methodology for simulation testing and confirmation of best practices in educational design for stressful environments. Although we focus on the use-case of untrained bystanders requiring instantaneous learning, this methodology may provide an additional avenue for clinical applications, such as instantaneous learning required by medical professionals. This extends to the design of instructions for medical equipment, not only for use in the field, but also in clinical settings where instructions need to be provided rapidly and accuracy is key. Furthermore, this study may be used to guide the development of a medical drone that is intended to deliver naloxone to opioid overdose situations for bystander administration. The analysis of instantaneous learning under acute stress can be directly applied to the user interface of the drone that provides instructions to bystanders. Our findings may contribute to instructions that result in successful administration of naloxone and improved clinical outcomes. Future studies are needed in other populations and with different methods of distraction to confirm the full applicability of the methodology of the present study. Also in future studies, completion time of each task may be used as an outcome measure to examine the impact of a stressful environment on the time it takes to learn instantaneously.

## Data Availability

All data generated or analyzed during this study are included in this published article.

## Abbreviations

AED: Automated external defibrillator
EMS: Emergency medical services
CPR: Cardiopulmonary resuscitation

## References

[1] Centers for Disease Control and Prevention (CDC). Opioid Overdose [Internet]. cdc.gov. 2020. Available from: https://www.cdc.gov/drugoverdose/index.html

[2] Giglio RE, Li G, DiMaggio CJ. Effectiveness of bystander naloxone administration and overdose education programs: a meta-analysis. Inj Epidemiol [Internet]. 2015;2(1). Available from: https://injepijournal.biomedcentral.com/articles/10.1186/s40621-015-0041-8 10.1186/s40621-015-0041-8 PMC5005759 27747742

[3] National Institute on Drug Abuse (NIDA). Naloxone Drug Facts [Internet]. drugabuse.gov. 2021. Available from: https://www.drugabuse.gov/publications/drugfacts/naloxone

[4] Doe-Simkins M, Walley AY, Epstein A, Moyer P (2009). Saved by the nose: bystander-administered intranasal naloxone hydrochloride for opioid overdose. Am J Public Health.

[5] Fischer P, Krueger JI, Greitemeyer T, Vogrincic C, Kastenmüller A, Frey D (2011). The bystander-effect: a meta-analytic review on bystander intervention in dangerous and non-dangerous emergencies. Psychol Bull.

[6] Faul M, Aikman SN, Sasser SM (2016). Bystander intervention prior to the arrival of emergency medical services: comparing assistance across types of medical emergencies. Prehospital Emerg Care.

[7] Kagan O (2018). Development of a measure to assess factors associated with college students’ willingness and readiness to Act in a food allergic emergency (WilRAFAE): a pilot. Cogent Psychol.

[8] Rudland JR, Golding C, Wilkinson TJ (2020). The stress paradox: how stress can be good for learning. Med Educ.

[9] Lighthall NR, Gorlick MA, Schoeke A, Frank MJ, Mather M (2013). Stress modulates reinforcement learning in younger and older adults. Psychol Aging.

[10] de Berker AO, Tirole M, Rutledge RB, Cross GF, Dolan RJ, Bestmann S (2016). Acute stress selectively impairs learning to act. Sci Rep.

[11] Dunham S, Lee E, Persky AM (2020). The psychology of following instructions and its implications. Am J Pharm Educ.

[12] Baddeley AD, Hitch G. Working memory. Psychol Learn Motiv - Adv Res Theory. 1974 Jan 1;8(C):47–89.

[13] Brooks LR (1967). The suppression of visualization by reading. Q J Exp Psychol.

[14] Brooks LR (1968). Spatial and verbal components of the act of recall. Can J Psychol Can Psychol.

[15] Dong X (2020). The general public’s ability to operate automated external defibrillator: a controlled simulation study. World J Emerg Med.

[16] Andre AD, Jorgenson DB, Froman JA, Snyder DE, Poole JE (2004). Aotomated external defibrillator use by untrained bystanders: can the public-use model work?. Prehospital Emerg Care.

[17] Attard Biancardi MA, Spiteri P, Attard J, Debono M, Mifsud J, Farrugia AB (2020). CPR performance in lay people with telephone assisted CPR instructions – a prospective manikin-based observational study. Malta Med J.

[18] Ettl F, Fischer E, Losert H, Stumpf D, Ristl R, Ruetzler K (2021). Effects of an automated external defibrillator with additional video instructions on the quality of cardiopulmonary resuscitation. Front Med.

[19] Yang T. The role of working memory in following instructions. 2011;(October).

[20] Kohn M, Senvak J. Sample size calculators for designing clincial research [Internet]. UCSF CTSI. 2021. Available from: https://www.sample-size.net/

[21] Wuensch K. Cohen’s conventions for small, medium, and large effects. Wuensch’s Statistics Lessons; 2019.

[22] Gulrajani N. DURI Conference Presentation [Video] [Internet]. YouTube. 2021. Available from: https://www.youtube.com/watch?v=OuSUUcEWaak

[23] Nahari G, Ben-Shakhar G (2013). Primacy effect in credibility judgements: the vulnerability of verbal cues to biased interpretations. Appl Cogn Psychol.

